# The Role of the BMI ≥ 40 kg/m^2^ Criterium in ASA-PS Classification for Metabolic Surgery

**DOI:** 10.1007/s11695-025-08118-7

**Published:** 2025-07-29

**Authors:** Elisabeth S. van Ede, Simon W. Nienhuijs, Marc P. Buise, R. Arthur Bouwman

**Affiliations:** 1 Department of Electrical Engineering, Signal Processing Systems, University of Technology, Eindhoven, The Netherlands; 2 Department of Anaesthesiology, Catharina Hospital, Eindhoven, The Netherlands; 3 Department of Surgery, Catharina Hospital, Eindhoven, Malta; 4 Department of Anaesthesiology and Pain Medicine, Maastricht University Medical Center, Maastricht, The Netherlands

**Keywords:** Metabolic surgery, Obesity, ASA-PS classification, Risk assessment

## Abstract

**Background:**

This study explores historical trends in ASA-PS scoring and evaluates whether the ASA-PS III classification based solely on a BMI ≥ 40 kg/m^2^ effectively contributes to peri-operative risk stratification for patients undergoing metabolic surgery.

**Methods:**

Adult patients (January 2015–January 2023) were included from the Dutch Audit for the Treatment of Obesity registry. Logistic regression adjusted for confounders compared groups stratified by BMI and presence of systemic disease. Outcome measures: complication prevalence (peri-operative, ≤ 30 days and > 30 days), Clavien-Dindo score, hospital stay (> 2 days), ICU admission, readmission, and mortality rates.

**Results:**

A total of 75,871 patients (78.8% women, age 43.5 (SD 11.8) years, BMI 42.9 (SD 5.3) kg/m^2^) were included. The number of patients receiving ASA-PS III increased from 26 to 92% (BMI ≥ 40 kg/m^2^) and from 20 to 64% (BMI < 40 kg/m^2^), despite generally stable mean age, BMI, and concurrent diseases. From 2018, ASA-PS II was inaccurately assigned in 64%. Patients without severe systemic disease (BMI ≥ 40 kg/m^2^) were significantly less likely to experience complications, ICU admission, prolonged hospital stay, or readmission compared to patients with severe systemic disease (BMI < 40 kg/m^2^) (OR: 1.72, 95% CI: 1.43–2.08, *p* < 0.001).

**Conclusions:**

A large Dutch national registry on metabolic surgery showed that ASA-PS III patients with BMI ≥ 40 kg/m^2^ only were less likely to experience complications after surgery compared to those with BMI < 40 kg/m^2^ and severe systemic disease. This suggests that adhering to the BMI criterion may undermine the ASA-PS classification’s effectiveness in risk stratifying these patients.

## Background

Since its introduction in 1941, the American Society of Anesthesiologists Physical Status (ASA-PS) score classification system has been used to classify the patient’s physical condition to assess the level of peri-operative risk. Although an increased BMI was added as a criterion in 2014, higher ASA-PS scores were often assigned to patients living with obesity in previous years prior to this update, potentially on theoretical or subjective grounds [1, 2]. However, the question remains whether assigning higher ASA-PS classifications solely based on a BMI ≥ 40 kg/m^2^ is evidence-based and accurately reflects peri-operative risk for patients undergoing metabolic surgery. Metabolic Surgery in this group is divided into two techniques: Roux-en-Y gastric bypass and gastric sleeve.

Living with obesity is linked to metabolic abnormalities heightening the risk of diabetes mellitus type 2, cardiovascular disease, or even death [3, 4]. Therefore, obesity-related complications are an important part of metabolic surgery indications and peri-operative risk assessment. BMI alone is still the most commonly used measure to estimate the degree of obesity and its associated metabolic diseases. Currently, patients with a BMI ≥ 40 kg/m^2^ are assigned the same ASA-PS classification as those with a BMI less than 40 kg/m^2^ along with the presence of concomitant diseases, suggesting a comparable cardio-metabolic vulnerability. Yet, there are different obesity phenotypes, of which not all patients exhibit metabolic disturbances [5, 6]. This so-called metabolically healthy obesity is characterized by less visceral adipose tissue and less inflammation, and a better cardiorespiratory fitness than patients with metabolically unhealthy obesity [5, 7]. It is also associated with a lower risk of developing cardiovascular disease [5, 8]. Some patients with metabolically healthy obesity will eventually develop metabolic syndrome and become metabolically unhealthy obese [5, 8]. Contributing factors are unique per individual and include male sex, a certain genetic profile for fat distribution, sedentary lifestyle, older age, higher total body fat content, and longer exposure to adipose tissue [7–13].

Although the severity of obesity and the presence of obesity-related complications can be independent risk factors for surgical blood loss, wound and urinary tract infections [14–16], increased BMI by itself cannot imply the presence of obesity-related complications, nor can it estimate the occurrence of peri-operative complications [17–20]. Also, for anaesthesia, obesity-related peri-operative risks are probably due to metabolic disease, such as myocardial infarction [16], rather than to technical limitations caused by an increased amount of adipose tissue, for example, a larger neck circumference [21] Previous research showed that compared with non-obese patients, more than or equal to class-II obesity was not associated with higher peri-operative morbidity or mortality after general surgery [14, 18, 19]. However, there is a lack of investigation into the impact of a BMI ≥ 40 kg/m^2^, as per the BMI criterion for a higher ASA-PS classification. Additionally, there is a notable absence of differentiation between groups based on the presence or absence of (chronic) cardiac or metabolic diseases. Furthermore, in a high-volume centre specializing in the care of patients living with obesity, the influence of solely BMI on peri-operative complications may be less significant.

Besides providing insight into the behaviour of the ASA-PS classification following the introduction of the BMI criterion, this study aims to test the hypothesis that ASA-PS III class only based on a BMI ≥ 40 kg/m^2^ exhibits lower complication or comorbidity rates compared to other patients in the same ASA-PS class after metabolic surgery and is thereby not contributing to peri-operative risk stratification.

## Methods

### Data Sources

Data in this retrospective study was derived from the Dutch Audit for Treatment of Obesity (DATO) [22]. DATO is a nationwide mandatory quality register since 2015. It collects detailed information from 20 centers of expertise in the Netherlands on patient, comorbidity, treatment, follow-up, and short- and long-term outcome characteristics of patients undergoing metabolic surgery. Details of the DATO regarding data collection, quality, and validation have been described elsewhere [22]. The DATO’s scientific committee unanimously approved the use of the data for this study (reference number DATO-2022–174). The study was carried out in accordance with ethical standards for research integrity and the regulations of the Dutch Institute for Clinical Auditing. No informed consent was required because this is an opt-out quality registry and is performed in accordance with the ethical standards of Dutch law. A data analysis and statistical plan was written after the data were accessed.

### Aims and Outcome Measures

To align with the aims of this study, three analyses were conducted. First, the behavior in the application of the ASA-PS classification was assessed. Second, the scoring ASA-PS compliance was evaluated. Third, the odds of morbidity and mortality were compared between two groups of patients assigned an ASA-PS III score: those classified solely based on a BMI ≥ 40 kg/m^2^ and those classified based on a BMI < 40 kg/m^2^ with the presence of severe systemic comorbid conditions.

### Data Inclusion

**Fig. 1:**
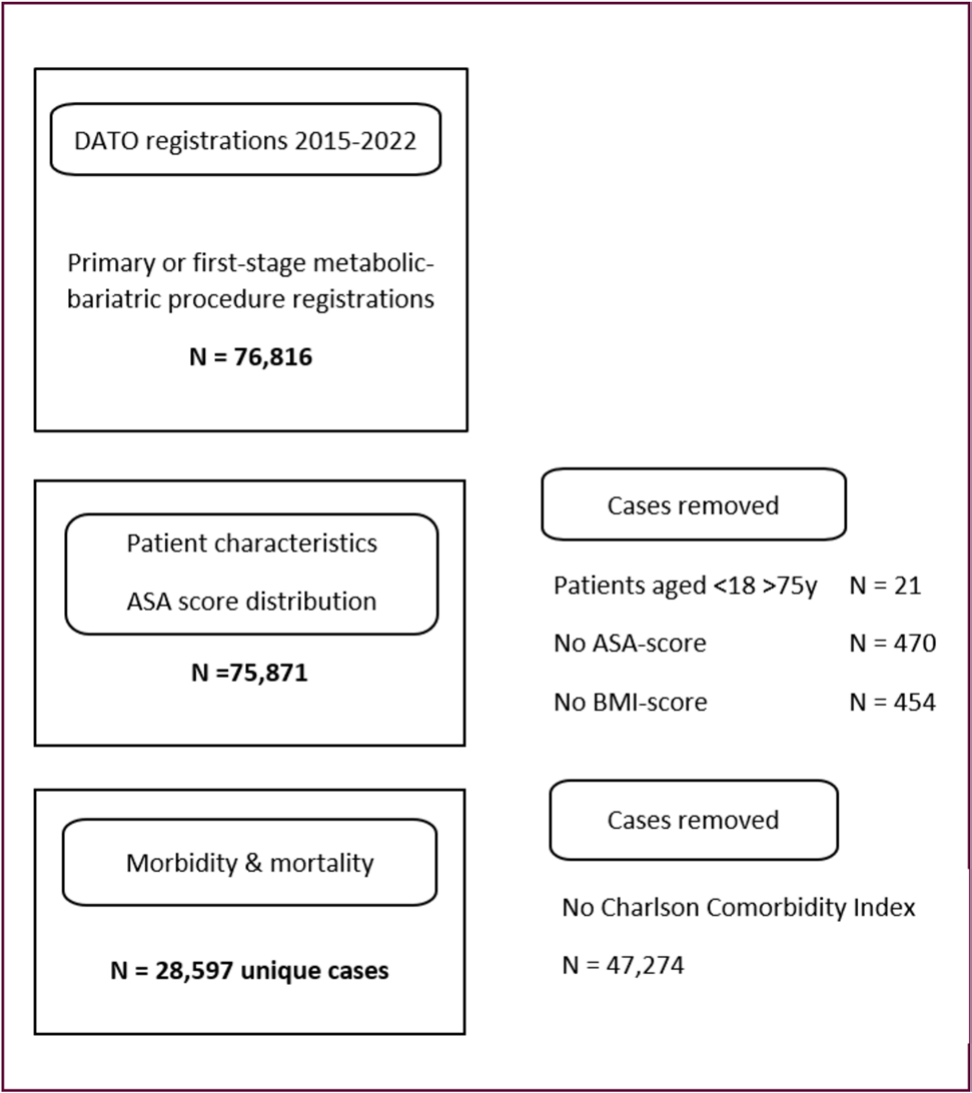
Case selection flowchart

Figure 1 shows a complete overview of data selection. General selection criteria included: age < 75 years, registered BMI ≥ 40 kg/m^2^ or a BMI ≥ 30 kg/m^2^ with an obesity-related comorbidity, registered ASA-PS classification, and primary or first of two-stage metabolic surgery. The included centers follow standardized metabolic surgery and anesthesia practices in alignment with Enhanced Recovery After Surgery (ERAS) guidelines [23]. Analyses of ASA-PS classification behavior included procedures between January 1 st 2015 and January 1 st 2023. Analysis of scoring compliance and morbidity and mortality included procedures between January 1, 2018, and January 1, 2023. This timeframe was selected to ensure the availability of a complete Charlson Comorbidity Index [24], which was used to define the study groups and was not reliably available in the data prior to 2018. Cases with missing information on comorbidities, ASA-PS score, BMI, and incomplete components of the Charlson Comorbidity Index were excluded from the study data (Fig. 1). Furthermore, patients with incomplete data on very specified peri- or postoperative complications were not excluded from the morbidity and mortality analyses.

### Definition of Study Groups

Patients were categorized into 3 groups based on their BMI level and severity of the concomitant systemic disease: (1) BMI 30–40 kg/m^2^, no/mild systemic diseases (group one), (2) BMI ≥ 40 kg/m^2^, no/mild concomitant systemic diseases (group two), and (3) BMI < 40 kg/m^2^, severe systemic diseases (group three). The latter two groups reflect patients who should have received an ASA-PS score III by the current guideline. The first group received an ASA-PS score II. For this study, severe systemic disease encompassed elements of the Charlson Comorbidity Index [24]. The components that should be all well-controlled before availability for surgery were for this study categorized as mild and included: non-metastatic malignancies, dementia, gastric ulcer, paralysis, HIV, connective tissue disease, uncomplicated diabetes mellitus 2, hypertension, obstructive sleep apnea syndrome, gastric esophageal reflux disease, musculoskeletal pain, and dyslipidemia.

### Definition of Outcomes

Mortality was defined as any death related to the registered procedure. Morbidity was defined as any complication to each unique patient case. Additional outcome measures involved the cumulative count of recorded complications, encompassing cases with more than one complication registry: prevalence of complication severity differentiated by Clavien-Dindo scores, prolonged hospital stays (> 2 days) [25], readmission rates, and ICU admission rates (planned and unplanned). If available, the prevalence of specifically peri-operative, short-term surgical and general (< 30 days after surgery), and long-term complications (> 30 days after surgery) was analyzed. Table S1 shows an overview of the included complication details. To test our hypothesis, statistical analyses will be performed between specifically groups II and III.

### Statistical Analysis

Data of patient characteristics were presented as mean with SD if normally distributed and as median with IQR if not normally distributed. Categorical data were described in an absolute number and or percentage. Differences within group characteristics were analyzed using ANOVA for numerical parametric, Kruskal–Wallis for numerical non-parametric data, and Chi-squared or Fisher’s exact test for binary data. Then, a multivariable regression model was used to analyze statistical differences of morbidity and mortality outcome measures between groups II and III. Potentially confounding variables were included in the model if considered clinically relevant and if a significant difference between groups was observed. Tested confounding variables included age, sex, waist circumference, and surgery type. The variables were tested for multicollinearity and effect modification. Group characteristic difference analyses were performed using R 4.2.1 and R studio (version 22.07.02 build 576) software, logistic regression analyses using SPSS software version 29.0.1.0 (171).

## Results

In total 75,871 patients (78.8% female) were included in the cohort for analyses of ASA-PS classification distribution. Their mean age was 43.5 (± 11.8) years, BMI 42.9 (± 5.3) kg/m^2^, and waist circumference 128.7 (± 13.5) cm. Of these patients, 23% underwent Sleeve gastrectomy, 77% Roux-en-Y Gastric Bypass.

### Distribution ASA-PS Classification Behaviour

**Fig. 2:**
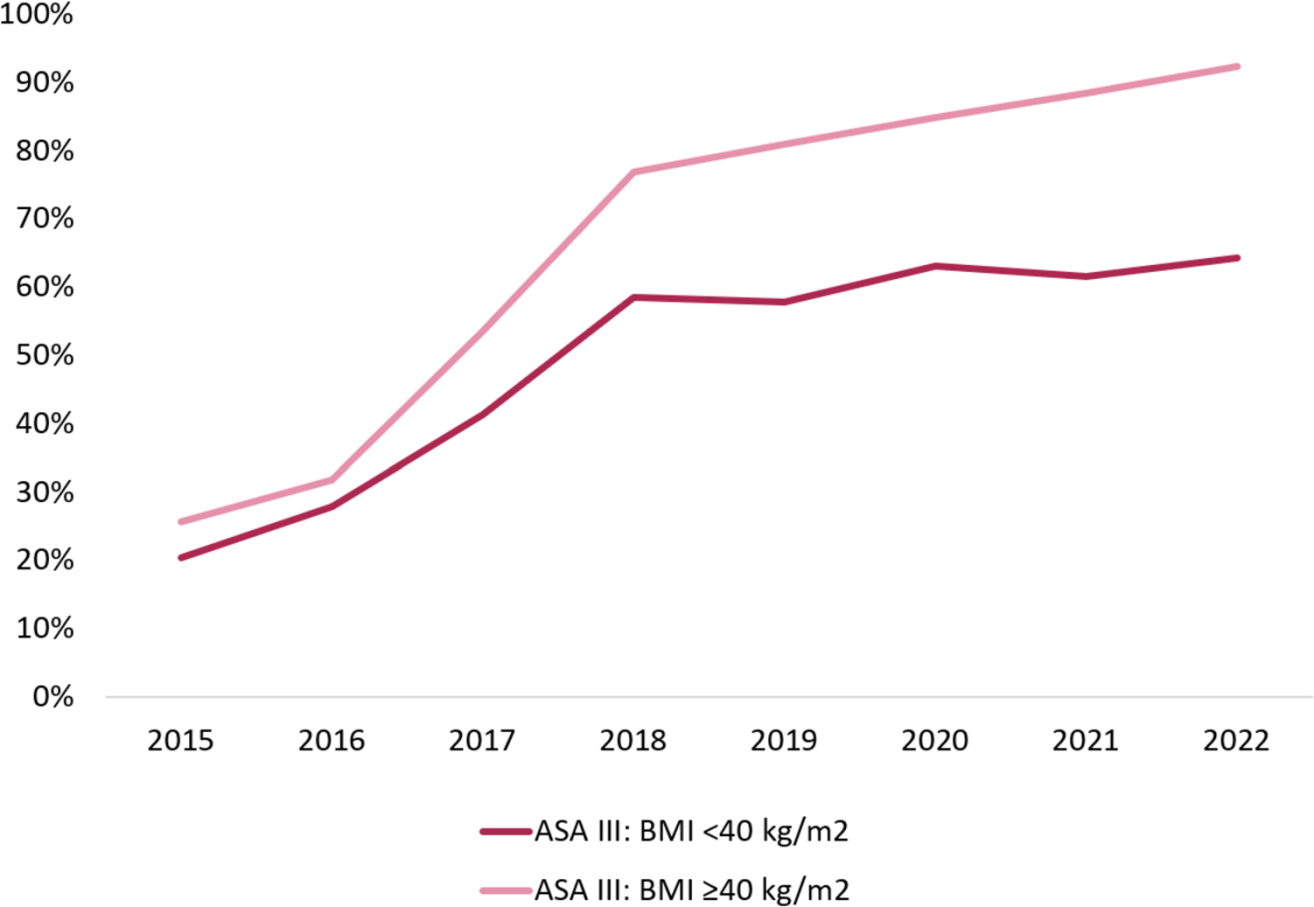
Distribution of ASA-PS classification 2015–2022. Percentage of patients with ASA-PS III distributed by BMI level (≥ 40 en < 40 kg/m^2^)

In 2015, 77% of the patients with a BMI < 40 kg/m^2^ and 70% with a BMI ≥ 40 kg/m^2^ received ASA-PS II (Fig. 2). These numbers decreased over time towards, respectively, 35% and 7% in 2022. Simultaneously, the number of patients scoring ASA-PS III increased for both BMI < 40 kg/m^2^ and BMI ≥ 40 kg/m^2^ levels (respectively 20% and 26% in 2015 and to 64% and 92% in 2022). These changes were most pronounced between 2015 and 2018.

The proportion of patients undergoing metabolic surgery with a BMI ≥ 40 kg/m^2^ decreased from 75.7% in 2015 to 68.6% in 2022. Patient characteristics remained largely consistent: in 2015, the average BMI was 43.5 ± 5.4 kg/m^2^ and the average age was 43.1 ± 11 years, while in 2022 their values were 42.6 ± 5.2 kg/m^2^ and 43.3 ± 12.3 years, respectively. Among patients with a BMI < 40 kg/m^2^, the prevalence of the sum of severe concomitant systemic disease decreased from 53% in 2018 to 26% in 2022 (Figure S1 for details).

### ASA-PS Classification Compliance

**Fig. 3:**
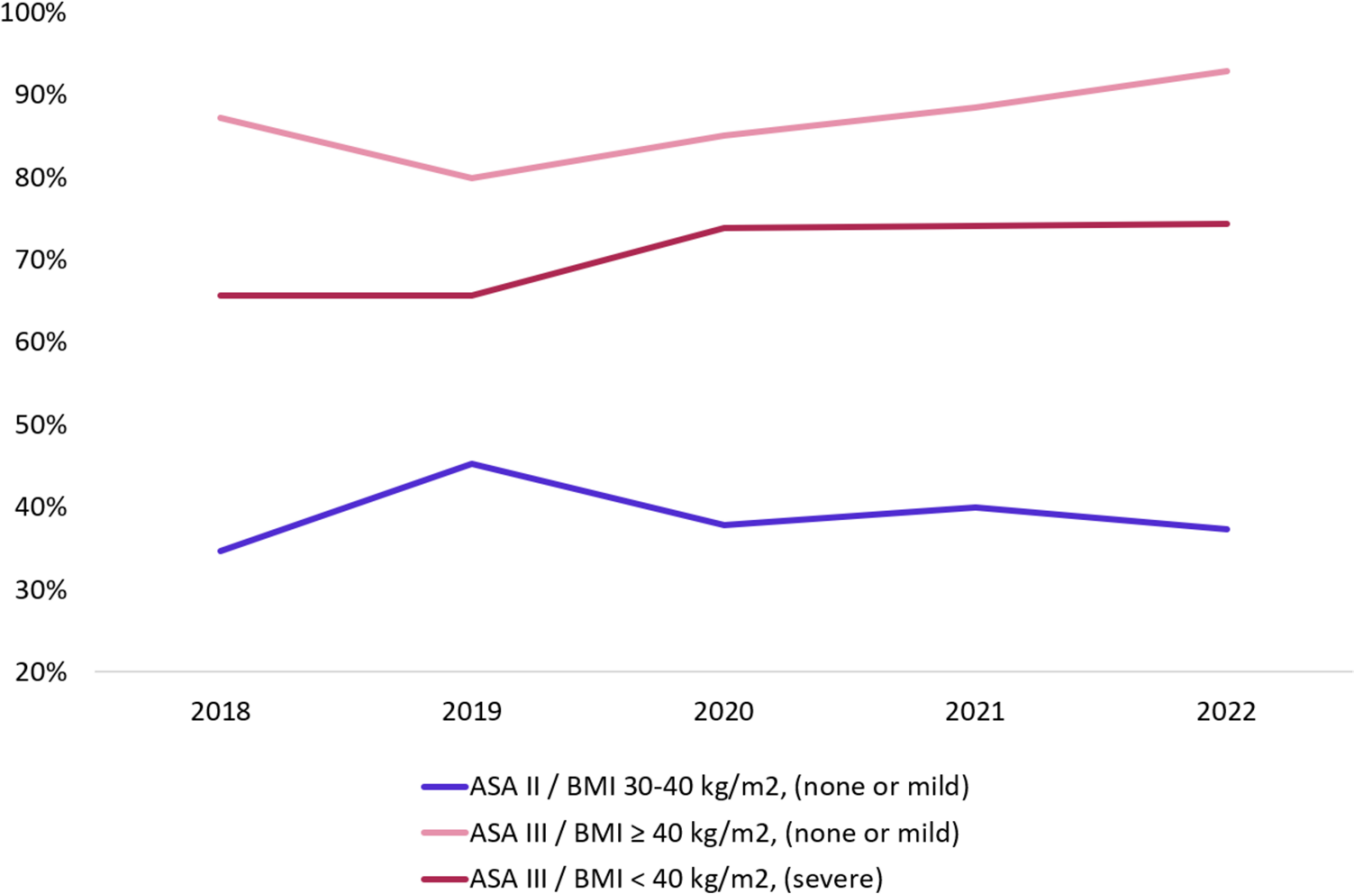
Compliance of ASA-PS classification 2018–2022. The percentage of patients that were accurately classified according to ASA-PS guidelines. None/mild/severe = concomitant disease status

In total 28,597 patient cases were available for this analysis (Fig. 1). From 2018 to 2023, an increased number of patients were assigned correctly ASA-PS III (averagely 77% in 2015 and 86.7% in 2022). Likewise, a decrease was encountered for patients receiving correctly ASA-PS II (averagely 41% in 2018 and 34% in 2022). The number of patients who received correctly ASA-PS III based on BMI ≥ 40 kg/m^2^ was even higher than the patients who received this on the basis of underlying severe systemic disease (in 2022 93% versus 74%) (Fig. 3).

### Morbidity and Mortality

Characteristics of the subgroups are shown in Table 1, the prevalence of morbidity and mortality in Table 2. The groups differed statistically from each other regarding age, waist circumference, BMI level, sex, and type of surgery (all *p* < 0.05). Of patients with none/mild systemic disease, regardless of BMI level, the number of patients with a complication was generally equal and even slightly lower for patients with BMI ≥ 40 kg/m^2^ (5.9% and 5.45%). This complication prevalence was highest for patients from group three (8.74%). This group had also the highest number of patients with more than one registered complication.

**Table 1:** Patient characteristics of the three groups and the group differences. Data presented as mean ± SD, or median [IQR]

	I	II	III	*p*-value
BMI level (kg/m^2^)	30–40	≥ 40	< 40	
Systemic disease	None/mild	None/mild	Severe	
Patients (*n*)	7887	18,068	2642	
Age (yrs)	46.4 ± 10.8	44.5 ± 12.9	50.2 ± 10.1	< 0.05
Waist circumference (cm)	120.8 ± 9.8	132.4 ± 13.7	122.4 ± 9.4	< 0.05
BMI (kg/m^2^)	37.6 [36.4–39.0]	43.4 [41.4–46.7]	37.5 [36.2–38.8]	< 0.05
Gender (female)	78.6	77.0	71.5	< 0.05
Roux-en-Y-gastric bypass (%)	81.7	77.6	81.9	< 0.05

**Table 2:** Group differences morbidity and mortality. Results of statistical analysis are adjusted for confounding variables and presented in odds ratio (OR) and 95% confidence interval (CI)

Group	I	II	III	II vs III: OR 95% CI, *p*-value
Patients with complicated case	5.9	5.45	8.74	1.72 [1.43–2.08], *p* < 0.001*
*Patients with* > *1 complication*	6.9	8.43	11.3	
*Clavien-Dindo score*				
I	1.18	1.14	1.59	1.56 [1.01–2.42], *p* = 0.05
II	1.47	1.67	3.23	1.79 [1.31–2.44], *p* < 0.001*
III	3.05	2.67	4.13	1.82 [1.41–2.34], *p* < 0.001*
*ICU admission*				
Planned	0.13	0.23	0.57	2.61 [1.18–5.76], *p* = 0.02*
Unplanned	0.25	0.17	0.38	2.00 [0.81–4.91], *p* = 0.13
Mortality	0.03	0.01	0.04	1.36 [0.16–11.64], *p* = 0.78 (UA)
Prolonged hospital stay (> 2)	3.98	3.60	7.34	1.97 [1.60–2.43], *p* < 0.001*
* Readmission*				
Short term	2.26	2.26	3.41	1.76 [1.30–2.37], *p* < 0.001*
Long term	1.50	1.32	1.86	1.40 [1.03–1.91], *p* = 0.03*
Peroperative	0.46	0.33	0.83	2.43 [1.28–4.62], *p* < 0.001*
Short term	4.18	3.61	6.70	1.80 [1.45–2.23], *p* < 0.001*
Surgical^#^	3.39	2.76	5.33	1.86 [1.46–2.37], *p* < 0.001*
General^#^	0.88	0.85	1.55	1.58 [0.99–2.50], *p* = 0.05
Long term	1.81	1.51	2.50	1.64 [1.20–2.25], *p* = 0.002*

*UA*; unadjusted result

*Significant results

^#^Analysis in case of available data

After adjustment for confounding variables, patients from group two were significantly less likely to have a complicated course than patients from group three (OR 1.72 95% CI 1.43–2.08, *p* < 0.001). From the sum of all registered complications, this latter group was also significantly less likely to have a complication scored CD-II or III, a planned ICU admission, a prolonged hospital admission, and to be readmitted in the short and long term. Information regarding specific surgical and general complications was not available for 118 out of 1093 records for patients from group two and for 11 out of 265 records for patients from group three. Analyses of these specified complications indicated a lower likelihood for patients from group two to experience short- and long-term peri-operative and surgical complications.

## Discussion

This study revealed that patients undergoing metabolic surgery in the Netherlands from 2015 to 2023 were, irrespective of their BMI level, increasingly assigned an ASA-PS III score. Furthermore, it showed that in this selected population, patients with a BMI ≥ 40 kg/m^2^ without severe systemic disease showed a significantly lower likelihood of experiencing surgical complications during their metabolic surgery trajectory compared to patients with a BMI < 40 kg/m^2^ and severe systemic disease. Additionally, they exhibited reduced probabilities of prolonged hospital stay, readmission, planned, and unplanned ICU admission. These results confirmed our hypothesis.

ASA-PS classification is a simple and fast way to gain insight into the patient’s health status and estimate postoperative risk [26]. The increase in ASA-PS III scores can largely be attributed to changes in the guidelines: Without taking into consideration co-morbidity or physical status, patients with a BMI ≥ 40 kg/m^2^ switched from score II to III. Notably, also patients with a BMI < 40 kg/m^2^ increasingly received an ASA-PS III. We have objectified that between 2018 and 2023, patients were incorrectly assigned an ASA-PS III score in 60–70% of cases. These patients had a BMI below 40 kg/m^2^ and no severe concomitant systemic diseases. Even before 2018, the population did not suffer from concomitant diseases more frequently, which may suggest that the unjustified allocation had started earlier. Although the ASA-PS classification guidelines have been made as objective as possible, it is well known that it remains subject to interpretation [27–29]. We hypothesize that by raising awareness about criteria for determining an ASA-PS classification through the introduction of a new guideline, stricter assessments and subjective up-coding leading to higher scores have occurred. Although the context differs, this effect bears some resemblance to the Hawthorne effect in terms of behavioral changes in response to external factors [30].

The presence of a serious systemic disease appeared to have a greater impact on the occurrence of complications during a metabolic surgery procedure compared to a BMI > 40 kg/m^2^. Although both are categorized as ASA-PS III, the operative risk profiles as assessed by the ASA-PS classification were not identical. Considering the observation that the rate of complications among patients with a BMI > 40 kg/m^2^ closely mirrored that of patients with a lower BMI, it indicates insufficient evidence to support the BMI criterion in our study population. Using BMI > 40 kg/m^2^ as the sole criterion for ASA-PS III classification undermines the efficacy of risk stratification in clinical practice. Accurate ASA-PS classification at the individual level would guarantee more precise allocation of hospital resources, better decisions regarding inpatient or outpatient surgery and timing of hospital discharge, and facilitating the delivery of personalized care efficiently while maintaining safety standards. Moreover, incorrect ASA-PS classification can result in research outcomes or quality evaluations that present a skewed perspective, rendering them unsuitable for reliable interpretation and implementation in clinical settings. Previous research in patients undergoing metabolic surgery showed a minimal difference in complications between ASA-PS II and ASA-PS III patients [31]. This could be explained by the fact that the results were based on the assigned ASA-PS classification and not on the actual underlying disease thereby reflecting clinical practice inaccurately.

BMI has frequently faced criticism as a metric of obesity severity or the risk of comorbidities or complications [32–34]. Instead, it is rather a static representation of a patient’s body composition at a given point in time, while, for example, long-term exposure to excess adipose tissue appears to be crucial in the development of obesity-related complications [9, 35–37]. Therefore, the inquiry arises regarding the suitability of the BMI metric for assessing health status altogether. Functional status or cardiorespiratory fitness, for instance, a Metabolic Equivalent Score, is also widely used in preoperative screenings. It is a measure of the amount of oxygen the body consumes during an activity compared to a resting state [38] and can be used to estimate whether the patient is able to withstand the physical demands of surgery regardless of age, body composition, or comorbidities [39]. Functional status is correlated logically with the ASA-PS classification, as illness could lead to immobility and vice versa; however, functional status has also been shown to be a better predictor than the ASA-PS classification itself [31]. It would be interesting to further explore the effect of incorporating functional status into the ASA-PS classification, instead of or in addition to BMI, as there are already indications that the latter is an independent predictor of mortality within each ASA-PS classification [40].

The recategorization of patients for concurrent systemic diseases was dependent on a diagnosis code, which was not always distinctive in terms of severity of the condition. For instance, hypertension encompassed both treated stable hypertension and untreated unstable hypertension, posing a potential risk of underestimating research results. In addition, data regarding smoking status or alcohol use was not available for analyses. Although these factors may not directly differentiate between ASA-PS II and III classifications, they should be taken into account as they may increase the risk of postoperative complications independently [41, 42]. These factors, along with the possibility of missing data, inconsistencies between centers, and potential coding errors, are inherent limitations of retrospective data collection. Additionally, the opt-out nature of the registry could introduce selection bias. However, no active opt-out requests have been made since the registry began. Despite these limitations, the data collection method remains closely aligned with current clinical practice, where the ASA-PS classification is not fully comprehensive.

Due to the limited number of cases with multiple complication registrations, a multilevel analysis was not performed. This could lead to repeated-measure bias, affecting the accuracy of effect size estimation. Notably, both the highest complication prevalence and the majority of patients who experienced more than one complication were identified in the subgroup characterized by BMI < 40 kg/m^2^ and severe systemic disease. Given the direct link between repeated measurements and complications in this context, the exclusion from multilevel analysis can be considered less problematic.

This study was strengthened by the large size of the cohort. The results are representative of Dutch clinical practice and may also be applicable to other countries with similar patient population levels of surgical selection or preparation and, importantly, a comparable healthcare system. The homogeneity of the cohort reduces the risk of bias in this setting.

We suggest that future research should investigate the relationship between increasing BMI and peri-operative complications to determine whether different BMI thresholds may offer greater accuracy. Also, our hypotheses could be examined in other cohorts undergoing metabolic surgery or in patients undergoing other surgery types.

Finally, although beyond the scope of this study, exploring other metrics that might serve as better indicators of health status and risks for patients living with obesity would be valuable to consider. Examples for more accurate health status assessment for patients living with obesity might include: degree of adiposity (e.g., BMI level and/or waist circumference), duration of BMI [43, 44], and the presence of tissue dysfunction or limitations in daily functioning (clinical obesity) [45]. With respect to the ASA-PS classification, it remains debatable whether incorporating such detailed assessments is feasible or appropriate, given its purpose as a rapid and pragmatic screening tool. Hypothetically, the clinical and physical consequences of adiposity—such as tissue dysfunction and reduced metabolic equivalent of task (MET) scores—may offer a more dynamic reflection of a patient’s functional reserve and could be considered either within or alongside the ASA-PS classification.

We conclude that due to changed guidelines, patients undergoing metabolic surgery in the Netherlands were increasingly assigned ASA-PS III, irrespective of BMI or concomitant systemic disease suggesting subjective upcoding. However, patients from this ASA-PS class based on solely a BMI ≥ 40 kg/m^2^ were less likely to have a complicated course after metabolic surgery than patients with a severe systemic disease. This finding suggests a devaluation of the value of ASA-PS in peri-operative risk stratification in patients living with obesity by sticking to a sole rigid criterium. It has significant implications for enhancing peri-operative resource utilization and improving efficiency without compromising safety. Correcting the overestimation of ASA-PS classification in patient assessments can not only contribute to more accurate risk stratification but also improve the overall validity of research results incorporating ASA-PS classification.

## Data Availability

No datasets were generated or analysed during the current study.
